# Integrated Traditional Chinese and Western Medicine for Chronic Spontaneous Urticaria: Protocol for a Trials Within Cohorts Study

**DOI:** 10.2196/88251

**Published:** 2026-08-04

**Authors:** Linhao Li, Sihan Wang, Jiale Chen, Xiaojie Ding, Yifei Wang, Fei Guo, Xiaoying Sun, Liu Liu, Xin Li

**Affiliations:** 1 Department of Dermatology, Yueyang Hospital of Integrated Traditional Chinese and Western Medicine, Shanghai University of Traditional Chinese Medicine, 110 Ganhe Road, Hongkou District, Shanghai, China, 86 18817773736; 2 Institute of Dermatology, Shanghai Academy of Traditional Chinese Medicine, Shanghai, Shanghai, China; 3 Shanghai University of Traditional Chinese Medicine, Shanghai, China

**Keywords:** protocol, clinical trial, chronic spontaneous urticaria, traditional Chinese medicine, omalizumab

## Abstract

**Background:**

Chronic spontaneous urticaria (CSU) is a common inflammatory skin disease characterized by severe itching and wheals, affecting approximately 1.4% of the global population. Both second-generation H1-antihistamines and omalizumab have limited efficacy in some patients. Previous studies suggest that combining traditional Chinese medicine with biological agents may improve efficacy and reduce adverse effects in inflammatory skin diseases.

**Objective:**

This study uses a trials within cohorts (TwiCs) design to evaluate the efficacy and safety of the *shenqi* formula (SQF) combined with omalizumab for the treatment of CSU.

**Methods:**

This TwiCs study is built upon an existing urticaria cohort established in November 2019 at Yueyang Hospital of Integrated Traditional Chinese and Western Medicine, Shanghai University of Traditional Chinese Medicine. Overall, 92 eligible patients with type 1 autoallergic CSU and *qi* and blood deficiency syndrome will be randomly allocated in a 1:1 ratio to receive either omalizumab alone or omalizumab combined with the SQF. The treatment period is 24 weeks, followed by 16 weeks of follow-up. The primary outcome is the 7-day Urticaria Activity Score. Data will be managed using Microsoft Excel (version 2016), analyzed with SPSS (version 27.0; IBM Corp), and visualized with GraphPad Prism (version 9.0). A complier average causal effect analysis will be applied to estimate causal effects using generalized linear latent mixed models.

**Results:**

As of the manuscript submission (November 2025), the trial has been funded (December 2024) and registered with the International Traditional Medicine Clinical Trial Registry (registered January 28, 2025). Patient enrollment commenced in April 2025 and is projected to conclude by December 2027. Data analysis is expected to be completed by June 2028, with final results anticipated for publication in the fourth quarter of 2028.

**Conclusions:**

This study is the first to apply a TwiCs design to compare the efficacy and safety of SQF combined with omalizumab versus omalizumab alone in patients with CSU. The findings are expected to provide high-quality real-world evidence for the integration of traditional Chinese medicine with biological therapy in the management of CSU and to serve as a methodological reference for future TwiCs applications in dermatology research.

## Introduction

Urticaria is an immune-mediated skin disease characterized by the development of wheals, angioedema, or both [1]. It is predominantly categorized into acute urticaria and chronic urticaria based on the duration of the disease manifestation. Chronic spontaneous urticaria (CSU) refers to a form of chronic urticaria that persists or recurs for more than 6 weeks, arising from known or unknown causes without an identifiable external trigger [2]. The etiology of CSU is heterogeneous. On the basis of distinct pathogenic mechanisms, CSU can be classified into allergic (type 1) and autoimmune subtypes (type 2b) [3,4]. The lifetime prevalence of chronic urticaria worldwide is 1.4%. In the Asia-Pacific region, the prevalence is 1.5%, with over 90% of cases being CSU [5].

Second-generation H1-receptor antagonist antihistamines are recommended as the first-line treatment for CSU [1,2,6]. Although second-generation H1-antihistamines are effective in many patients, the response can be poor and the improvement unsatisfactory, restricting clinical applications. Moreover, immunoglobulin E (IgE)–mediated inflammatory responses are pivotal in the development and progression of CSU [7]. Therefore, IgE-targeting therapy can be more effective when H1-antihistamines are not sufficient to control CSU. Omalizumab, a recombinant humanized anti-IgE monoclonal antibody, is currently one of the most mature and widely used IgE-targeting therapies [8]. Multiple clinical studies have confirmed that omalizumab can significantly reduce the 7-day Urticaria Activity Score (UAS7) and decrease the number of wheals [9]. However, some studies found that the efficacy of omalizumab alone in treating CSU remains between 55% and 73% [10-12]. After treatment with omalizumab, patients still experience issues such as easy relapse and adverse reactions. Hence, it is urgent to continue to seek treatment options for patients with CSU.

The earliest documented treatment of urticaria in traditional Chinese medicine (TCM) can be traced back to the *Waitai Miyao*, with a history of more than 1300 years. TCM has been applied for urticaria clinically in China for a long period. Clinical studies have confirmed that TCM has advantages in alleviating symptoms of urticaria [13-16]. The *shenqi* formula (SQF) is an empirical prescription from Xia’s family in TCM surgery, which is composed of 12 TCM ingredients (Table 1). Our research team has found that the SQF is safe and effective in treating CSU through long-term clinical practice. Several studies indicate that combining TCM with targeted therapies can enhance efficacy and reduce relapse rates in dermatological conditions [17,18]. However, there is a lack of clinical research confirming the efficacy and safety of SQF combined with omalizumab for CSU. Therefore, we adopted a trials within cohorts (TwiCs) study design, a derivative of a randomized controlled trial (RCT) design [19], to evaluate the efficacy and safety of the combination therapy.

**Table 1.** The component of the *shenqi* formula.

Chinese name	Latin name	Medicinal parts	Dosage in the trial (g)
*Huang qi*	*Astragali radix*	Root	30
*Dan shen*	*Salviae miltiorrhizae radix et rhizoma*	Root and rhizome	30
*Sheng di huang*	*Rehmanniae radix*	Root	30
*Yi yi ren*	*Coicis semen*	Seed	30
*E zhu*	*Curcumae zedoariae rhizoma*	Rhizome	27
*Xu chang qing*	*Cynanchi paniculati radix et rhizoma*	Root and rhizome	20
Chi Shao	*Paeoniae Rubra Radix*	Root	9
Chao Bai Zhu	*Atractylodis Macrocephalae Rhizoma Praeparata*	Rhizome	9
Fang Feng	*Saposhnikoviae Radix*	Root	9
Jing Jie	*Schizonepetae Herba*	Herb	9
Chuan Xiong	*Chuanxiong Rhizoma*	Rhizome	9
Huang Qin	*Scutellariae Radix*	Root	9

TwiCs is a derivative design based on the principles of RCTs, in which multiple randomized comparisons can be embedded within an existing prospective cohort. This design is particularly well-suited for evaluating integrative medicine approaches such as the combination of TCM with biologics. First, TwiCs allows for the observation of intervention effects in a real-world clinical setting, where patient preferences and long-term medication adherence are naturally reflected factors that are especially relevant in TCM practice and where treatment often involves complex herbal formulations and requires sustained patient cooperation. Second, by embedding the trial within a well-established cohort, this design facilitates long-term follow-up and repeated measurements, which are essential for assessing both the durability of treatment response and the safety of combined therapy over time. Third, the TwiCs design enables the evaluation of multiple interventions within the same cohort infrastructure, offering an efficient platform for future studies that may compare different TCM formulas or combination strategies. Therefore, this design not only preserves the internal validity of a randomized trial but also enhances the external validity and practicality of findings in the context of integrative dermatology.

## Methods

### Study Setting

**Figure 1.**
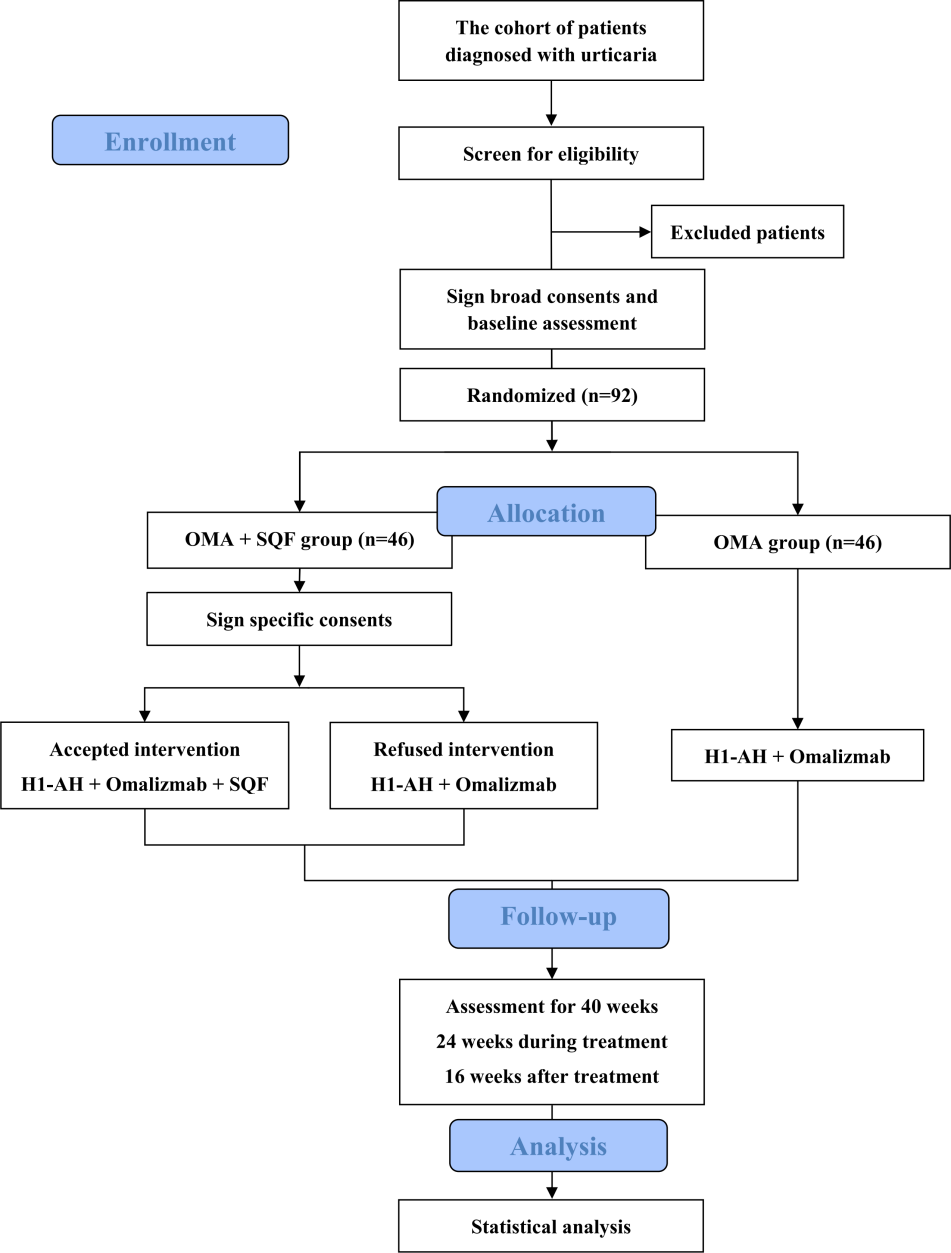
Flowchart of recruitment, randomization, and follow-up of the study participants. OMA: omalizumab; SQF: *shenqi* formula.

This is a cohort-based trial in which we have established a cohort of patients with urticaria at Yueyang Hospital of Integrated Traditional Chinese and Western Medicine, Shanghai University of Traditional Chinese Medicine. The urticaria cohort (established in November 2019) includes patients with CSU who have provided broad consent for future research. This broad consent covered two tiers: (1) consent for longitudinal observational follow-up and (2) consent to be considered for potential randomization into future low-risk intervention trials embedded within the cohort—a consent pattern that is well-documented in the TwiCs literature. From this cohort, we enrolled patients with type 1 autoallergic CSU and *qi* and blood deficiency syndrome who were treated with omalizumab in both inpatient and outpatient settings (Figure 1).

This 2-stage consent approach—broad cohort consent followed by trial-specific consent—explicitly differentiates between cohort participants providing control data and trial participants receiving the experimental intervention. Eligible patients with CSU will be enrolled in the study and randomly allocated to 2 groups at a 1:1 ratio: the omalizumab group (control group) and the omalizumab combined with SQF group (intervention group). Unlike a conventional RCT in which all participants are informed of randomization before enrollment, the present study is embedded within a preexisting CSU cohort with prior broad consent for future research participation. Eligible participants are identified from the cohort and randomized within the cohort infrastructure. The intervention group receives additional consent for SQF treatment, while participants receiving routine omalizumab-based care continue standard follow-up procedures within the cohort framework. Therefore, this study preserves key TwiCs features, including cohort embedding, staged consent, and real-world treatment evaluation.

### Ethical Considerations

Ethics approval was granted by the institutional review board of Yueyang Hospital of Integrated Traditional Chinese and Western Medicine, Shanghai University of Traditional Chinese Medicine (2024‐238). The trial has been registered at the International Traditional Medicine Clinical Trial Registry (ITMCTR2025000308). This protocol was developed according to the Standard Protocol Items: Recommendations for Interventional Trials 2025 guidelines [20,21]. After ethics approval of the protocol, eligible patients from the cohort will be recontacted. Those who provided prior broad consent will be invited to sign a trial-specific informed consent form detailing the SQF intervention, potential risks, and their right to withdraw at any time.

### Diagnostic Criteria

The diagnosis of type 1 autoallergic CSU is based on a comprehensive assessment of the patient’s medical history, symptoms, signs, and auxiliary examinations [1]: (1) symptoms and signs include dispersed wheals of varying sizes, angioedema, or both, and often accompanied by itching; (2) there are no clear triggers before the onset; (3) the condition lasts for at least 6 weeks with recurring episodes; and (4) serum total IgE level must be elevated above the upper limit of the normal.

Patients who meet all of the following criteria can be diagnosed with *qi* and blood deficiency syndrome for *yinzhen* in TCM [22,23]: (1) The condition recurs repeatedly and persists for months or even years without resolution; (2) pale red wheals that worsen after fatigue, accompanied by itching, tiredness, and insomnia with vivid dreams; (3) pale and swollen tongue with a thin coating; and (4) soft and thin pulse.

### Eligibility Criteria

Patients who meet all of the following recruitment criteria can be included in the study: (1) they meet the clinical diagnostic criteria for type 1 autoallergic CSU and *qi* and blood deficiency syndrome in TCM; (2) they are aged between 18 and 65 years, with no gender restrictions; (3) they volunteer to participate in this study and sign the informed consent form; (4) they are currently receiving treatment with omalizumab; (5) they have received omalizumab for no more than 3 months; and (6) they have been on a stable, standard-dose regimen of a second-generation H1-antihistamine, cetirizine, for at least 4 weeks before study entry.

Patients who meet any of the following criteria will be excluded: (1) other skin diseases or active conditions that may affect the evaluation of efficacy, such as angioedema or lupus erythematosus, are present; (2) they have severe mental illness or cognitive impairment, lack personal autonomy, or are unsuitable for clinical research participation; (3) they are currently experiencing severe, uncontrollable acute or chronic infections; (4) they have severe systemic diseases or clinical test results meeting any of the following conditions: alanine aminotransferase or aspartate aminotransferase levels elevated by more than 1.5 times the normal value; serum creatinine levels elevated by more than 1.5 times the upper limit of the normal value; any of the major blood routine parameters (white blood cell count, red blood cell count, hemoglobin level, platelet count) below the lower limit of the normal range; and other laboratory test abnormalities, in which the investigator deems the patient unsuitable for participation in the study; (5) they have a history of malignant tumors, primary or secondary immune deficiencies, or hypersensitivity; (6) they have undergone major surgery within the past 8 weeks or are expected to undergo such surgery during the study period; (7) they are pregnant or breastfeeding; (8) they have a history of alcohol abuse, drug addiction, or substance abuse; (9) there are contraindications for the use of omalizumab; and (10) there are other reasons that may make the patient unsuitable for the study.

### Randomization and Allocation

The random allocation sequence will be generated using a computer-based random number generator by an independent statistician not involved in the trial. The generated allocation codes will be placed in sequentially numbered opaque, sealed envelopes prepared by the same statistician. After an eligible participant is enrolled and has signed the informed consent form, a research nurse who is unaware of the allocation sequence will open the next envelope in order. The participant will then be allocated to either the omalizumab group or the omalizumab + SQF group according to the code inside the envelope. The entire allocation sequence will remain concealed from all investigators and participants until the moment of envelope opening. All patients in the SQF group will now receive full disclosure of the herbal ingredients at the time of consent.

### Sample Size

Sample size calculation is based on the sample size calculation method for TwiCs [19]. According to previous research, the improvement rate of the 7-day UAS7 after omalizumab treatment of CSU was 63.1% (*P*=.63) [24], while the improvement rate after omalizumab combined with TCM herbal formulation intervention was 84.6% (*P*=.85) [25]. The significance level (α) and the test power were set as .05 and 0.9, respectively, corresponding to β=1−0.9=0.1. For the 2-sided test, 42 participants would be needed for each group. Assuming a 10% dropout rate following the intervention, the required sample size is 46 patients per group (control and experimental), resulting in a total sample of 92 participants:


$$n=\frac{p_{1}\times \left(1-p_{1}\right)+p_{2}\left(1-p_{2}\right)}{{\left(p_{2}-p_{1}\right)}^{2}}\times {\left(\mu_{\alpha /2}+\mu_{\beta }\right)}^{2}$$

Since no prior human study has directly evaluated SQF combined with omalizumab in CSU, effect size estimation was partially informed by preclinical evidence demonstrating immunomodulatory effects relevant to IgE-mediated inflammation [24,25]. Therefore, the current sample size should be considered exploratory and hypothesis generating.

### Interventions

Patients will be randomly assigned to 1 of 2 groups: omalizumab or omalizumab combined with SQF. Participants in both groups will receive basic treatment and omalizumab injection.

For basic treatment, all participants will receive second-generation H1-antihistamines [1,2,6], including bilastine, cetirizine, desloratadine, ebastine, fexofenadine, levocetirizine, and rupatadine. The specific medication will be determined by the physician based on the patient’s condition and previous medication history. The medication will be administered at the recommended dosage stated in the package insert, taken orally once daily. Additionally, physicians are allowed to adjust antihistamine dosage up to 4 times compared to the standard dose based on the patient’s condition during the treatment. Any dosage adjustments will be thoroughly documented in the case report form.

For omalizumab injection, omalizumab for injection (XOLAIR [Novartis Pharma Stein AG]; 150 mg in a single-dose vial, approval number S20170042) will be administered subcutaneously in the deltoid area of the patient’s upper arm at a dose of 300 mg per session, once every 4 weeks [26].

Additionally, the intervention group will be treated with SQF orally. The SQF is manufactured by Shanghai Wanshicheng Medicines Co Ltd. It is processed into 200-ml bags of herbal liquid, packaged in medical-grade plastic preservation bags by the company. The herbal medicine liquid is heated and taken orally, twice a day, with 1 bag per dose (Table 2).

**Table 2.** Study timetable of the trials within cohorts design. Drug adjustment of treatment in week 4: H1-antihistamines can be gradually reduced for patients in the omalizumab group if improvement is observed after 4 weeks of treatment. Early termination of treatment in week 14: treatment will be terminated in patients in the omalizumab group if no improvement is observed after 12 weeks of treatment.

		Screening and enrollment	Treatment						Follow-up	
		Week 0	Week 2	Week 4	Week 12	Week 14	Week 16	Week 24	Week 32	Week 40
Enrolment										
	Informed consent	✓								
	Eligibility screen	✓								
	Baseline record	✓								
	Randomization and allocation		✓							
	Lesion photograph	✓	✓	✓	✓	✓	✓	✓	✓	✓
Interventions^a^			✓	✓	✓	✓	✓	✓		
Assessments										
	UAS7^b^	✓	✓	✓	✓	✓	✓	✓	✓	✓
	Traditional Chinese medicine syndrome score	✓	✓	✓	✓	✓	✓	✓	✓	✓
	IgE	✓			✓			✓		✓
	UCT^c^	✓	✓	✓	✓	✓	✓	✓	✓	✓
	CU-Q2oL^d^	✓	✓	✓	✓	✓	✓	✓	✓	✓
Safety assessments										
	Vital signs	✓	✓	✓	✓	✓	✓	✓	✓	✓
	Drug combination	✓	✓	✓	✓	✓	✓	✓	✓	✓
	Blood routine	✓			✓			✓		✓
	liver and kidney function	✓			✓			✓		✓
	Adverse events		✓	✓	✓	✓	✓	✓	✓	✓
	Serious adverse events		✓	✓	✓	✓	✓	✓	✓	✓
Others										
	Drug adjustment			✓^e^	✓					
	Early termination					✓^f^				

a Interventions: H1-antihistamines + omalizumab for omalizumab group; H1-antihistamines + omalizumab + SQF for omalizumab + SQF group.

b UAS7: 7-day Urticaria Activity Score.

c UCT: Urticaria control test score.

d CU-Q2oL: Chronic Urticaria Quality of Life Questionnaire.

e H1-antihistamines can be gradually reduced for patients in the omalizumab group if improvement is observed after 4 weeks of treatment.

f Treatment will be terminated for patients in omalizumab group, if no improvement is observed after 12 weeks of treatment.

### Outcome

#### Primary Outcomes

The primary outcome is the UAS7 [27]. The primary time point for the comparison of the primary outcome is at week 40. UAS7 reflects the disease activity of the patient with urticaria over the past week and tracks the patient’s urticaria-related symptoms and signs over 1 week. It records both the number of hives and the severity of itching on a daily basis. The UAS7 also evaluates the patient’s condition daily based on 2 aspects: the objective number of hives and the subjective severity of itching. Both are graded on a scale of 0 to 3, with the scores ranging from 0 to 3 points [28]. These scores will be used to assess the activity of urticaria during the week before the follow-up and to monitor changes in the patient’s condition.

#### Secondary Outcome

Secondary outcome measures include total serum IgE levels, the Urticaria Control Test (UCT), the Chronic Urticaria Quality of Life Questionnaire (CU-Q2oL), and the TCM syndrome score.

Total serum IgE levels will be obtained by drawing venous blood from patients and analyzing it in the laboratory. UCT reflects the long-term control of urticaria in patients [29]. It assesses the overall disease status, the frequency of urticaria symptom episodes, the impact on quality of life, and the frequency of disease episodes that are difficult to control with medication over the past 4 weeks [30]. The scoring uses a 0 to 4 scale for each of these 4 dimensions, with lower scores indicating better urticaria control. CU-Q2oL comprehensively evaluates the quality of life in patients with chronic urticaria [31]. The questionnaire consists of 23 items, where patients self-assess the impact of 4 factors—“sleep problems,” “daily activities,” “limitations,” and “symptoms”—on their lives over the past 2 weeks [32]. The questionnaire uses a 5-point Likert scale, converting the patients’ responses into scores ranging from 0 to 100 [33]. The TCM syndrome score refers to the grading and quantification of symptoms associated with *qi* and blood deficiency, as outlined in the “Guidelines for Clinical Research of New Chinese Medicines (Trial)” [34]. A scoring scale for chronic urticaria with *qi* and blood deficiency syndrome was developed, evaluating clinical symptoms, tongue appearance, and pulse condition. The TCM syndrome score uses a 0 to 3 grade scoring system, where higher scores indicate more severe manifestations of the TCM syndrome in patients [35].

### Safety Assessments

Safety indicators include vital signs (temperature, respiratory rate, heart rate, and blood pressure in the examination room), concomitant medications, laboratory test results (blood routine, liver and kidney function), adverse events, and serious adverse events. The researchers will monitor adverse events and serious adverse events throughout the study. In case of adverse events, the researchers have prepared contingency plans for their management and will provide treatment as appropriate. In the case of serious adverse events, the researchers will immediately take action to ensure participant safety, promptly report the event to the principal investigator and the ethics committee, and determine whether the participant should be withdrawn based on the situation. All information related to adverse events or serious adverse events and the corresponding actions will be documented in the patients’ case report forms in detail.

### Dissemination Plan

The results of this trial will be disseminated through peer-reviewed publications in open-access journals, presentations at international dermatology and allergy conferences, and a plain-language summary for patients and health care providers.

### Statistical Analysis

Data registration and management will be performed using Microsoft Excel (version 2016), and statistical analysis will be conducted with SPSS software (version 27.0; IBM Corp). Graphical representation will be done using GraphPad Prism (version 9.0).

A complier average causal effect analysis will be applied [36]. This analysis divides the participants into 4 categories based on their assigned and actual treatments: compliers, never-treated participants, always-treated participants, and always-treated participants with the opposite treatment. The complier average causal effect focuses on compliers and estimates the causal effect for this group, fitting the data using a generalized linear latent mixed model. Antihistamine dose changes during the trial will be recorded and included as a covariate in a sensitivity analysis to assess their potential impact on the primary outcome.

Categorical data will be described using rates. For continuous data, if normally distributed, means and SDs will be used for statistical description. If the data are not normally distributed, medians and IQRs will be used. For normally distributed data with homogeneity of variance, independent samples 2-tailed *t* tests or 1-way ANOVA will be applied. Nonparametric tests will be used for nonnormally distributed data. Comparisons of rates will be conducted using the chi-square test, with pairwise comparisons among multiple groups performed using chi-square splitting. A *P* value of <.05 will be considered statistically significant.

## Results

Participant recruitment was initiated in April 2025. As of manuscript submission (November 2025), the cohort comprised 188 patients. Patient enrollment is projected to conclude by December 2027. Of the 188 patients who had been screened for eligibility, 48 participants met the inclusion criteria and 46 participants were enrolled and randomized according to the study protocol. Follow-up assessments are ongoing, and no serious adverse events related to the intervention have been observed to date. Data analysis is expected to be completed by June 2028, with final results anticipated for publication in the fourth quarter of 2028.

## Discussion

### Summary of Anticipated Findings

This TwiCs trial is expected to demonstrate that add-on SQF therapy improves urticaria control compared to omalizumab alone, with a clinically meaningful reduction in the UAS7 at week 16. We also anticipate that the combination therapy will be well tolerated, with no significant increase in adverse events.

### Rationale for the Combination Therapy

CSU is a skin disease that can have a long duration and recur over time. CSU causes edema-like skin lesions and can affect sexual function [37], as well as causing anxiety, depression, or fatigue [38]. Moreover, the majority of patients have insomnia due to the severe itching, which seriously affects their physical and mental health [39]. Over 20% of patients experience a daily impact on their ability to work [40]. The treatment costs for CSU exceed 900 purchasing power parity dollars annually [41], imposing a significant financial burden on patients. The biological agent omalizumab is widely used in clinical practice, but its effectiveness is limited to less than 70% [42]. The pathogenesis of CSU allows for its classification into 2 main subtypes: an allergic form, driven by IgE-mediated hypersensitivity to unseen allergens, and an autoimmune form, characterized by autoantibodies against Fc epsilon R1A (FCER1A) or IgE [43,44]. Clinical research has shown that low serum IgE levels correlate with a significantly reduced response to omalizumab [45,46]. Its adverse reactions, such as cough, headache, upper respiratory tract infection, or gastric ulcer, also cannot be ignored [47]. Patients with CSU urgently need more effective and safe treatments.

SQF is the classical formula from Xia’s family in TCM surgery and is effective in clinical application. SQF is suitable for patients with CSU of the *qi* and blood deficiency type. Its functions are dispelling wind, nourishing blood, replenishing *qi*, supplementing blood, strengthening the spleen, and transforming dampness. Pharmacological studies show that the ingredients in the formula, such as *astragali*, *atractylodis*, *saposhnikoviae*, *scutellariae*, and *paeoniae*, possess anti-inflammatory and immune-regulating effects through stabilizing mast cells and reducing IgE levels [48]. Current research suggests that mast cells play a central role in the pathogenesis of urticaria, and IgE-mediated inflammatory responses are pivotal in the development and progression of CSU. IgE binds to its receptor FCER1, activating mast cells [49]. Then activated mast cells release inflammatory mediators, leading to the development of urticaria [7]. *Astragali* and *salviae*, the main herbs in SQF, are proven to have a targeted effect on mast cells and IgE. *Astragali* can reduce IgE and histamine secretion, inhibit mast cell degranulation, and then stabilize mast cells [50,51]. Moreover, *salviae* works by dampening FCER1 signaling [52]. Then the IgE stimulation in mast cell activation is prohibited. Considering the targeted effects of omalizumab and SQF on IgE and mast cells, this study combines them to evaluate whether the combination enhances efficacy.

### Interpretation of the TwiCs Design

TwiCs is an innovative cohort multiple RCT research method first proposed by Relton et al [53]. In general, the TwiCs design is based on a prospective cohort in which all the participants provide informed consent to receive treatment and provide data for adjusting medications. In addition, an important feature of this method is that multiple RCTs can be conducted within a single cohort. This research takes into account the medication of patients with CSU, and is expected to improve treatment adherence. On the other hand, the natural progression of CSU and the dynamic changes following intervention (SQF or omalizumab) can be continuously monitored. Moreover, this is the first TwiCs study to compare the efficacy and safety of SQF plus omalizumab in the treatment of CSU. We hope this study can provide high-quality evidence for TCM combined with biological agents in CSU treatment, provide more management options for CSU, and offer a reference for the application of TwiCs design in clinical research in dermatology.

The TwiCs design adopted in this study has important implications for how the findings should be interpreted. First, regarding generalizability, TwiCs is embedded within an existing prospective cohort that reflects real-world clinical practice, where patients receive background treatments according to routine clinical protocols rather than rigid study mandates. This design feature enhances external validity, meaning that the results are more likely to be applicable to real-world settings where patients may have varying disease courses, concomitant medications, and treatment preferences. Second, concerning noncompliance, TwiCs is particularly well-suited to address treatment adherence challenges that commonly occur in real-world practice, especially in TCM where herbal formulations may affect acceptability.

Although the TwiCs design offers notable methodological advantages, several limitations should be acknowledged. Selection bias in cohort recruitment is a potential concern, as the cohort was established at a single tertiary hospital. Patients who seek care at this institution may differ systematically from those treated elsewhere in terms of disease severity, socioeconomic status, or treatment expectations, which could affect the representativeness of the study population. Another limitation is the open-label design, as patients in the intervention group are aware of receiving SQF in addition to omalizumab. Although outcome assessors may remain blinded, the lack of participant blinding may introduce performance bias, particularly for patient-reported outcomes such as UAS7 and quality of life measures.

### Strengths and Limitations of the Study

This study adopts a derivative design called TwiCs, making the trial process more reflective of real-world clinical practice.

Multiple outcome measures (UAS7, IgE levels, UCT, Chronic Urticaria Quality of Life Questionnaire [CU-Q2oL]) are used to assess efficacy from both subjective and objective perspectives, which ensures the reliability of the trial results.

**Figure 2.**
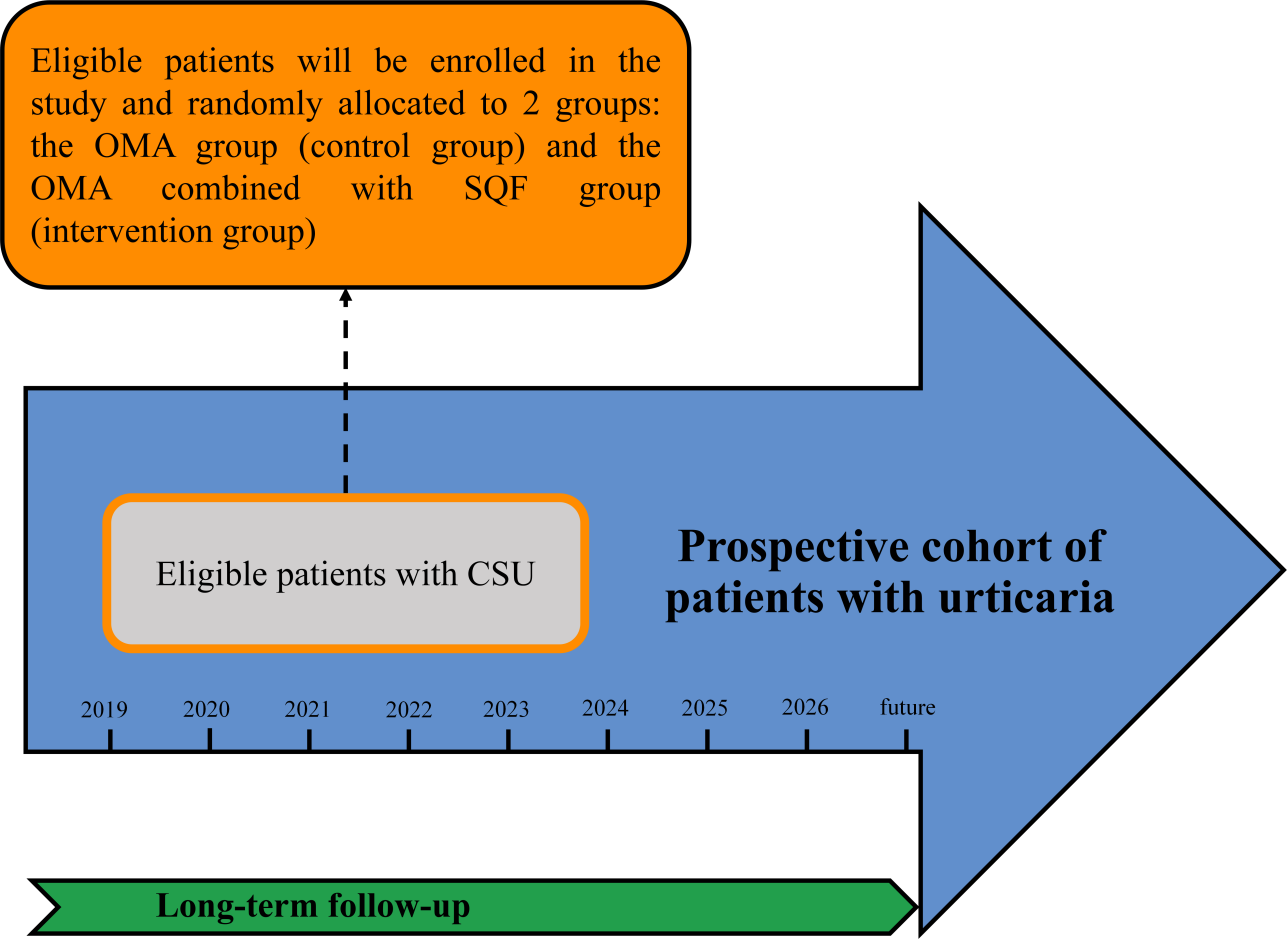
Overview of the course of the trials within cohorts design. CSU: chronic spontaneous urticaria; OMA: omalizumab; SQF: *shenqi* formula.

This study is the first TwiCs method to compare the efficacy and safety of SQF and omalizumab in the treatment of CSU (Figure 2).

This study is conducted exclusively at a single hospital, which may restrict the generalizability of the findings to broader populations.

### Future Directions

If the anticipated benefits are confirmed, future research should consider (1) a multicenter TwiCs design to improve generalizability, (2) biomarker analyses to identify which CSU subtypes derive the greatest benefit from add-on SQF, and (3) longer-term follow-up to assess durability of response and relapse rates after treatment discontinuation.

## Supplementary material

10.2196/88251 Checklist 1 SPIRIT 2025 checklist.

## Abbreviations

CSU: chronic spontaneous urticaria
CU-Q2oL: Chronic Urticaria Quality of Life Questionnaire
FCER1: Fc epsilon R1
IgE: immunoglobulin E
RCT: randomized controlled trial
SQF: *shenqi* formula
TCM: traditional Chinese medicine
TwiCs: trials within cohorts
UAS7: 7-day Urticaria Activity Score
UCT: Urticaria Control Test
